# Let's face it: reading acquisition, face and word processing

**DOI:** 10.3389/fpsyg.2014.00787

**Published:** 2014-07-23

**Authors:** Paulo Ventura

**Affiliations:** Faculty of Psychology, University of LisbonLisboa, Portugal

**Keywords:** reading acquisition, neuronal recycling, faces, words, holistic processes

## A tight link between reading acquisition and changes in face processing

The invention of writing is one of the most important cultural changes of mankind. Notably, because reading was invented only 5000 years ago, there was not sufficient time to evolve a brain system devoted to visual word recognition. Nevertheless, learning to read leads to the development of a strong response to written materials in the left fusiform gyrus, in the “visual word form area” (VWFA, e.g., Dehaene, 2009). Consequently, reading must rely on pre-existing neural systems for vision and language, which may be partially “recycled” for the specific problems posed by reading (Dehaene, 2005; Dehaene and Cohen, 2007). This consistent localization is related to prior properties of the corresponding tissue, which make it particularly suitable to the specific problems posed by the invariant visual recognition of written words (Dehaene, 2009): bias for foveal stimuli (Hasson et al., 2002), posterior-to-anterior increase in perceptual invariance (Grill-Spector et al., 1998; Lerner et al., 2001), and more direct projection fibers to language areas (Cohen et al., 2000; Epelbaum et al., 2008).

The neuronal recycling model predicts that, as cortical territories dedicated to evolutionarily older functions are invaded by novel cultural objects, their prior organization should slightly shift away from the original function (though the original function is never entirely erased). As a result, reading acquisition should displace whichever evolutionary older function is implemented in the site of the VWFA. In a recent fMRI study (Dehaene et al., 2010) comparing illiterate to literate adults, we showed that learning to read competes with the cortical representation of other visual objects, especially faces. With increasing literacy, cortical responses to faces decrease slightly in the left fusiform region, while increasing strongly in the right fusiform area (FFA). Thus, right-hemispheric lateralization for faces is increased in literates compared to illiterates. Consistent evidence was also reported when comparing 9-year-old normal readers and dyslexic children. Not only did responses to written words showed a greater left lateralization in normal readers, but responses to faces were also more strongly right lateralized (Monzalvo et al., 2012; cf. also Monzalvo, 2011).

Further developmental studies also reveal a tight link between reading acquisition and changes in face processing. Cantlon et al. (2011) demonstrated that 4-year-old-children show decreasing responses to faces in the left fusiform gyrus with increasing knowledge of letter and number symbols. Li et al. (2013) found for Chinese preschooler's a facilitative effect of early exposure to reading in neural responses to visual words. Such a facilitative effect had a temporary cost in neural response to faces, which did not show the mature pattern seen in adults. Dundas et al. (2012) studied the development of hemispheric specialization for written words and faces in children, adolescents and adults, and found that the left-hemispheric specialization for words develops prior to the right-hemispheric specialization for faces, with face lateralization related to reading comprehension ability (cf. also Pinel et al., 2014).

The competition between different categories seems to rely on enhanced neuronal specificity, namely on decreased responses to non-preferred stimuli as opposed to an increased response to the preferred category. Indeed, Cantlon et al. (2011) found a decrease for non-preferred stimuli in VWFA and FFA during child development. Joseph et al. (2011) reported both *progressive changes*, i.e., increasing face-specialization in a brain region with age, and *regressive change*s, i.e., decreasing face-specialization with age. Indeed, many brain regions recruited in children for face processing showed reduced specialization for faces by adulthood. Such regressive changes support the idea that some areas of the face network may lose out, at a cellular and a functional level, to promote specialization. Consistent with this scenario, in Pinel et al. (2014)'s study stronger leftward asymmetry for the VWFA and rightward asymmetry for the FFA were characterized by reduced activation in homolog areas of the contralateral hemisphere.

All these observations support the existence of competition for cortical space between the VWFA and the pre-existing neural coding of faces (Dehaene, 2005; Dehaene and Cohen, 2007; Plaut and Behrmann, 2011), with some displacement of fusiform face-sensitive areas toward the right hemisphere. A similar theoretical perspective (Nestor et al., 2012; Behrmann and Plaut, 2013) espouses a competitive interaction between distributed circuits for faces and word recognition for foveally-biased cortex, constrained by the need to integrate reading with the language system primarily left-lateralized.

Recently, we investigated the behavioral consequences of this brain reorganization (Ventura et al., 2013). Using the face composite task, we found that literates are consistently less holistic than illiterate individuals without any reading experience. The effect is not even specific to faces, but extends to houses. It thus seems that literacy induces a shift in the ability to deploy analytic visual strategies, over and above any specific effect that it may have on face processing, at least in tasks requiring selective attention to part of an object: it reduces automatic reliance on holistic processing when it is detrimental to the task by enabling the use of a more analytic and flexible processing strategy (Ventura et al., 2013). These findings are in apparent contradiction with Lachmann et al's (2012) study of congruence effects for letters vs. geometric shapes. Illiterates displayed negative CEs for both letters and shapes a finding which was interpreted as indicative of a generic and primary analytic perceptual processing strategy that is not reading specific, on par with the holistic strategy. In a previous study (Ventura et al., 2008) using the Framed-Line-test, we showed that both illiterates and ex-illiterates use context dependent/holistic processing. These apparently contradictory results most probably result from a host of factors including characteristics of the different stimuli, different task demands, and different meanings of analytic and holistic in these different studies.

In sum, the studies reviewed show that the acquisition of reading has an extensive impact on the developing brain and reveal a tight link between reading acquisition and changes in face processing. In the following section I evaluate whether reading acquisition leads to words becoming an object of visual expertise with some processing characteristics similar to faces and other objects of expertise.

## Words as an object of visual expertise

Faces are made from common features (eyes, nose, mouth, etc.) arranged in the same general configuration. Thus, beyond the presence of specific features or the location of these features, subtle differences in facial features and their spatial relations are particularly useful for successful recognition of a given face (e.g., Maurer et al., 2002). To facilitate extraction of configural information people process faces holistically—as a whole—rather than as a collection of individual face features.

The many thousands encounters with visual words lead to a visual expert processing of these stimuli. Like in face processing, recognition of written words relies on both their features (e.g., letters) and the configuration between them. Recent work by Wong and colleagues adopting the sequential matching paradigm commonly used with faces showed HP to be a marker of expertise for perception of English words (Wong et al., 2011). Wong and colleagues also showed that HP of words was sensitive to amount of experience with the stimuli, with larger holistic processing for native English readers than Chinese readers who learned English as their second language. Holistic processing of Chinese characters also seems to develop as one acquires expertise (Wong et al., 2012). Indeed, expert Chinese readers displayed a larger holistic processing for characters than non-characters.

The larger holistic processing of words with reading experience seems in apparent contradiction with our evidence of smaller holistic processing of faces and houses (Ventura et al., 2013). This discrepancy may stem from differences in the processes that lead to the development of holistic processing in the face/object domain vs. word domain: fine subordinate-level discrimination among highly similar objects vs. the development with reading expertise of orthographic representations comprising multiple components that can be processed in parallel (Wong et al., 2012), enabling direct access to the lexicon. In this vein, it would be interesting to evaluate more directly the development of holistic processing of words as children develop reading proficiency. The beginning reader uses a letter-by-letter reading strategy. It is during this relatively slow process of phonological recoding that exposure to printed words enables the setting up of a specialized system for parallel letter processing (cf. Share, 1995; Grainger et al., 2012). One might predict a relationship between the development of this parallel orthographic processing and HP of words.

Although the same composite paradigm has been used to reveal HP for faces and words, this does not mean that the same mechanisms underlie the effects for the two domains (Chen et al., 2013). However, Chen et al. (2013) showed that HP of words has an early perceptual locus similar to that for faces. Nevertheless, the correlate of HP for characters was P1 (reflecting perceptual processing in extrastriate visual cortex) different from the N170 commonly found for face HP.

Reading is undoubtedly an expert visual function and these experiments show that word perception can be affected by holistic processes. However, there are fundamental distinctions between words and faces. The mature reading network comprises not only a visual shape analysis (VWFA system), but also components involved in print-to-sound translation and access to word meaning.

Considering print-to-sound translation, classic studies (e.g., Ziegler et al., 2000) have shown that upon seeing a written word both an orthographic and a phonological code are rapidly activated, although this last code lags slightly beyond the orthographic code. It is clear that the experiments of Wong and colleagues show HP for words: matching target parts of a word was interfered by the irrelevant parts, and such interference was reduced when the parts were misaligned. But this reduction with misalignment might result not (only) from disruption of visual configural processes, but (also) from a disruption of the phonological code. This question is the subject of ongoing research. One avenue is to compare computer vs. handwritten print. Letters in handwritten words are noisy, variable, and ambiguous and their physical forms are affected by neighboring letters and thus configural processes may assume a more prominent role in handwritten words (Barnhart and Goldinger, 2013). If the effects found in the word composite task are indeed due to configural visual processes, HP should be greater for handwritten words. If, however, the effects are, at least in part, due to disruption of phonological codes, there should be small differences between both types of words. Another avenue is to use words with letters that have different pronunciations (e.g., “rena,” dear, and “remo,” rowing, in which the “e” grapheme has different phonological values). If the effects reported above are purely visual, these different pronunciations should not influence the HP of words.

Another interesting point concerns the definition of the left and right parts of the (alphabetic) words used in the holistic paradigm. Written words have several linguistic units: syllables, onsets, and rimes, graphemes, and letters. In the experiments of Wong et al. (2011), for some stimuli the left-right division respects a division between two psycholinguistic units—onset and rime (e.g., cr|ew)—while for others the division straddles psycholinguistic units: onset and nucleus vs. coda (e.g., ki|ck). It would be interesting to compare systematically the holistic/configural effects for sets of words in which the left|right division respects a division between two psycholinguistic units vs. a left|right division that do no respect such a division. If the effects reported are indeed due to configural/holistic processes, they should occur even when the left|right division straddles psycholinguistic units.

In sum, words are psycholinguistics units comprising both perceptual and linguistic factors. The many thousands encounters with words by the typical literate person give rise to a perceptual expertise with effects similar to what has been observed for faces and other objects of expertise. However, further clarification of the origin of HP effects for words is needed.

In conclusion, reading acquisition leads to changes in face processing and extensive reading experience can result in expertise effects for words similar to what has been found for faces and other objects of face-like expertise.

### Conflict of interest statement

The author declares that the research was conducted in the absence of any commercial or financial relationships that could be construed as a potential conflict of interest.
